# The Treatment of Local and General Peritonitis

**Published:** 1890-06

**Authors:** W. E. B. Davis

**Affiliations:** Birmingham, Ala.


### THE TREATMENT OF LOCAL AND GENERAL PERITONITIS.

---

The above is the title of a paper read at the recent meeting of the Alabama Medical Association, by Dr. W. E. B. Davis, of Birmingham.

From a study of the experiments of Pawlowsky, Grawitz, Wegner and others, he thinks the following points pretty well settled:

*First.* Simple peritonitis, when caused by a sufficient quantity of a chemical irritant, will produce death by the extent of the inflammation.

*Second.* Simple inflammation may terminate in septic peritonitis, by producing a weakened condition of the walls of the intestines, which permit the passage of septic germs from the intestinal canal into the peritoneal cavity.

*Third.* While pathological germs in a small quantity may be absorbed by the healthy peritoneum, without producing a peritonitis, the same quantity combined with a chemical irritant may

*Op. .

produce a violent inflammation—the irritant having prevented the absorption of the germs and caused the exudation of a nutrient fluid for their multiplication.

*Fourth.* Large quantities of septic fluids and microbes always produce suppurative peritonitis; yet a small quantity of either may be absorbed and destroyed, unless the peritoneum has been weakened by antecedent pathological changes.

*Fifth.* A septic fluid may gravitate into dependent parts of the peritoneum, and become shut up, either by plastic inflammation, or by a coil of intestine, and thus be prevented from producing diffuse peritonitis, but after a time this may rupture and produce death from general peritonitis.

*Sixth.* The germs of septic peritonitis will be found in the kidneys and other organs of the body, and in greater quantities, according to the extent and duration of the inflammation.

*Seventh.* The condition of the peritoneum, and the nature and quantity of the septic product will determine the rapidity of the inflammation, which usually ends in from forty-eight hours to six days, but death may be produced from shock in a few hours. Tubercular inflammation is always slow in its progress.

From a consideration of the foregoing principles, he says the following indications for treatment must be arrived at:

1. Promote absorption of the inflammatory products of simple peritonitis as rapidly as possible, and thus relieve the inflammation and prevent the possibility of septic peritonitis.

2. In the early stage of peritonitis, whether simple or septic, where the cause cannot be determined, hasten the absorption of inflammatory products, etc., with purgatives.

3. When medical treatment fails to give relief, septic fluids should be removed by operative procedure.

4. In localized . peritonitis—with circumscribed pus formation—the pus should be removed and the abscess cavity drained.

5. In acute septic peritonitis, operative procedure must be adopted early or there will be no chance of recovery offered by the operation, as the inflammation will become more extensive the longer it continues, and, too, there will be so great a quantity of septic germs absorbed into the system, that death will result from toxæmia, even though the local inflammation should be remedied by a late operation.

He quotes from Habershon and others, and states that it has been demonstrated that, in the large majority of cases, peritonitis is a symptom of some well-recognized lesion of the apdominal or pelvic viscera, and that the only rational treatment must be based upon this conception of the disease. Peritonitis is not a "disease distinct," as taught by Bichat, and upon which teaching the treatment of Alonzo Clark gained such great popularity. The "opium splint" is irrational, for it not only locks up the products of inflammation, but as shown by Wylie, Johnson, Baldy and others, and by his own experience, subjects the patient to one of the greatest dangers of the disease, viz., obstruction of the bowels from adhesions.

In case of perforation of the bowel, opium is indicated to relieve pain and shock, and to prevent peristalsis and further escape of the intestinal contents into the peritoneal cavity. Again, morphine hypodermically may be used, with benefit, in some cases, when there is persistent and uncontrollable vomiting; but at the same time, calomel in small and frequently repeated doses, may be dropped on the tongue and the bowels induced to act. There are many cases in which it is absolutely necessary to give hypodermic injection for pain, but this should never be given in such doses as recommended by the advocates of the opium treatment, and should not be administered at all unless the patient's condition is being made more grave by the shock provoked from pain.

The first two indications for treatment are best met by free purgation, as taught by Tait and others, and the majority of those who have adopted this plan select the magnesium salts, as they produce very large watery stools. When the stomach rejects salts, calomel may be used.

He refers to a large number of cases treated by him in the most satisfactory manner by purgation—and among them several cases of threatened peritonitis after laparotomies. During the past year he has not waited for symptoms of peritonitis after a laparotomy, but begins the use of small doses of salts, and if not retained, of small doses of calomel, a few hours after the patient gets from under the influence of the anæsthetic, and aids the pur gative by the administration of enemas of milk and whiskey every third hour, which relieve thirst and stimulate and nourish the patient if retained.

In these cases he had to give an occasional hypodermic of morphine, but this did not prevent the bowels acting. He has had to depend on calomel oftener than salts, as it was not rejected. He reports cases illustrating how purgative treatment aids in diagnosis, and others to show how all symptoms may be masked by opium, and an operation delayed too long—and concludes by stating that it is very important not to resort to the free use of morphine, unless an operation has already been decided on, and this administered to relieve pain and lessen shock.

After cases of abortion, or delivery at full term, in addition to large doses of ergot, to produce rapid involution of the uterus, he has his patients, within twelve or fifteen hours, take a decided dose of salts to prevent peritonitis.

He reports a number of cases of perityphlitis, and advocates early operative interference. Since the cæcum and appendix are always completely invested with peritoneum, as demonstrated by Bull, the abscess of the appendix must be intra-peritoneal at the beginning, as has been shown by the experience of McBurney, Weir, Wylie and others.

He agrees with Wylie that should the symptoms of local peritonitis, in the region of the cæcum, not begin to improve by the fourth or fifth day from saline treatment, and local applications over the seat of the inflammation, an incision should be made down through the muscles and the peritoneum dissected up, until a place is found where the abscess is attached, and then opened. While the operation would be easier if delayed, the danger of the abscess rupturing and producing acute septic peritonitis must be borne in mind, and hence the increased difficulty in doing the operation is more than compensated for in the risk saved the patient.

Cases are reported to show that a negative result with the hypodermic needle should never cause a moment's delay in operating. He operates just as promptly when he can find no pus.

He endorses the views of those who advocate the removal of the appendix in frequently repeated attacks of appendicitis, for the same reason that he would remove the tubes and ovaries for recurring attacks of pelvic peritonitis, when they are the cause of the inflammation.

In acute septic peritonitis, as met with in child-bed fever, or after perforation of the bowels, or from the emptying of the contents of an abscess into the cavity, or after operative procedures, or accidental traumatism, such as gun-shot wounds, stabs, etc., nothing short of a laparotomy can afford any chance of recovery, and this will not offer much prospect unless done very early.

He quotes the experiments of Pawlowsky, and recites his own experiments on animals to demonstrate how rapidly septic peritonitis may be developed and produce death. He also reports a number of cases of gun-shot injuries and stabs of the intestine, in which he has seen violent attacks of peritonitis developed in a few hours, and in which the symptoms before operation did not indicate its development. Hence, when the abdominal cavity has been entered, it should be opened and explored immediately. To wait for symptoms is to wait too long. Give morphine to relieve pain and shock, and operate even though the patient should feel perfectly well after the relief thus afforded.

In cases of perforation of the appendix, etc., the operation should be done at once—unless the patient is almost pulseless—as by so doing the shock will be relieved. The same rule will hold in cases of ruptured abscesses.. In cases of perforation in typhoid fever, the condition of the patient before the accident must be taken into consideration, as pointed out by Mears; but as this is a fatal accident, unless it can be remedied by operative measures, this procedure should not be condemned without having been tried in a larger number of cases.

After a review of the open plan of treatment, as suggested by Dr. B. E. Hadra—which he condemns—he recommends the following method, which should be adopted in all cases of *acute general suppurative peritonitis*, and which will allow of the complete exposure of the abdominal cavity, the removal of the cause of inflammation, and assist in restoring the functions of the intestines.

The abdomen is opened in the median line; the cause, if found, removed; the cavity thoroughly douched with hot water; all adhesions broken up, and if tympanites is not marked, drainage tubes are introduced, through which the cavity may be washed out, as indications require. If the cause be found in the region of the cæcum, the drainage tubes should be introduced through a second incision in the right iliac region.

In those cases in which tympanites is marked, causing pressure on all the abdominal organs, and thus creating much constitutional trouble, it will require special attention, and upon this point he lays great stress; for this condition is a dangerous one of itself. Not only does the weakened intestinal wall permit of the continued passage of septic germs into the peritoneal cavity and afford constant infection, but it must be remembered that the bowel cannot be replaced without great pressure and consequent traumatism, which will often kill in a few hours from shock thus induced. In advanced cases of peritonitis it must also be remembered that the walls of the intestines are rendered inactive by inflammation, and the power of contraction cannot be restored until the inflammation is relieved; and hence, the bowel will continue tympanitic and the exchange of septic germs kept up, unless this condition is remedied. Dupaul punctured the intestine with a fine hollow needle in cases of tympanites, with dangerous pressure symptoms, and this has been recommended by the leading writers up to this time; even Senn refers to this as a procedure which may be resorted to. This has been tried by the author a number of times, and he was never able to see an appreciable decrease in the tympanites, and he argues that it is not reasonable to suppose that a paralyzed bowel could expel any quantity of gas through a needle. He has also practiced making incisions into the bowel, and by pressure attempted to expel the gas, but this does not prove satisfactory. He considers the best method of relieving a distended, paralyzed gut, full of poisonous gas, is to fill it with hot water, as this will not only free it of tympanites, but in getting rid of the gas and feces, etc., prevents infection.

Hence, in extreme cases, he believes an opening should be made in the lower part of the ileum, and the bowel thoroughly irrigated. An artificial anus should be formed, and the bowel irrigated through a soft tube as necessary to prevent tympanites and adhesions. Purgatives can have but little effect on a bowel, in fully developed septic peritonitis, when nearly all the coats are inflamed; so we must reach it mechanically. With this plan the colon can be washed through a rectal tube, the small intestine irrigated as required through the artificial anus, and the peritoneal cavity drained and douched.

This method meets all the indications for treatment, and is the one which should be adopted in all cases where marked tympanites is present.

